# Studying additive effects of combining rTMS with cognitive control training: a pilot investigation

**DOI:** 10.3389/fnhum.2023.1201344

**Published:** 2023-07-31

**Authors:** Iris Dalhuisen, Céline Schutte, Bob Bramson, Karin Roelofs, Philip van Eijndhoven, Indira Tendolkar

**Affiliations:** ^1^Department of Psychiatry, Radboud University Medical Center, Nijmegen, Netherlands; ^2^Donders Institute for Brain Cognition and Behavior, Radboud University, Nijmegen, Netherlands; ^3^Behavioral Science Institute, Radboud University, Nijmegen, Netherlands

**Keywords:** repetitive transcranial magnetic stimulation, cognitive control training, depression, neuronavigation, mood induction

## Abstract

**Background:**

Repetitive transcranial magnetic stimulation (rTMS) on the dorsolateral prefrontal cortex (DLPFC) is an effective treatment for depression that has been proposed to work via the enhancement of cognitive control. Cognitive control training (CCT) can also alleviate depression by relying on DLPFC activation. As the additive effects of rTMS and CCT are unclear, we set out to conduct a within-subject pilot study in healthy controls.

**Methods:**

Seventeen participants received two sessions of individualized resting-state connectivity-guided high-frequency rTMS, while randomly performing CCT or a control task. After each session, a negative mood was induced.

**Results:**

We found effects on mood and cognitive control after rTMS + CCT as well as rTMS + control, which were indiscriminative between conditions. Based on the statistical evidence for the absence of an additive effect of CCT, we did not perform a full study.

**Conclusion:**

Our results demonstrate no differential effects of single sessions combining rTMS and CCT in a healthy population, even with the methodological improvement of individualized neuronavigation. The improvement in cognitive control seen in both conditions could indicate that a simple cognitive task is sufficient when studying additive rTMS effects. Future studies should focus on augmenting the effects of various cognitive tasks and compare the present interventions with rTMS or cognitive tasks alone.

## 1. Introduction

Repetitive transcranial magnetic stimulation (rTMS) is used for treating depression at various stages of severity and has been shown to be effective. However, with response and remission percentages of ~30–60%, there is still room for improvement (Sackeim et al., 2020). The most common rTMS target for depression is the dorsolateral prefrontal cortex (DLPFC), which is part of the frontoparietal control network and has been defined as the functional basis of cognitive control (Miller, 2000; Ochsner and Gross, 2005; Carter and van Veen, 2007). An important theory regarding the mechanisms of action of rTMS concerns the neural modulation of these networks mediating cognitive control. Cognitive control is closely related to affective disorders because it plays a critical role in emotion regulation processes (Ochsner and Gross, 2005). Mood improvement after treatment with rTMS could be the result of improved cognitive control over emotion regulation processes. Indeed, studies have shown that high-frequency rTMS over the left DLPFC increases the excitability of this region (Fitzgerald et al., 2006; Schutter, 2010). Additionally, cognitive control has been shown to improve in both healthy participants and depressed patients after stimulating the DLPFC with rTMS (Corlier et al., 2020; Pulopulos et al., 2022). Importantly, the effect of stimulation depends on the state of the targeted brain region during stimulation (Sathappan et al., 2018). This state dependency can be controlled by functionally engaging specific neural circuits, for instance by combining rTMS with a cognitive task or therapy that targets the same neural network (Sathappan et al., 2018). Combining rTMS with cognitive tasks or therapies is highly feasible, and several studies have confirmed that therapeutic response in patients with MDD can be affected (Luber et al., 2008; Vedeniapin et al., 2010; Neacsiu et al., 2018). In this study, we will further elaborate on the potential options in the choice of cognitive task taking individualized brain stimulation into account as well as a cognitive task that has shown to have an augmented treatment effect in depression.

A logical choice to take advantage of the state-dependent effects of rTMS is cognitive control training (CCT), which is also used as a stand-alone treatment strategy for depression (Siegle et al., 2007, 2014) and results in increased activity in the frontoparietal network and other brain regions implicated in affective and executive function, notably the anterior cingulate cortex (Schweizer et al., 2013; Kim et al., 2017). In addition, the n-back task, which is often used in CCT, has been shown to robustly recruit the DLPFC (Owen et al., 2005).

Both rTMS and CCT are effective treatment strategies for depression, and they target and activate the DLPFC. Currently, patients with MDD are often passively treated with rTMS. We reasoned that the therapeutic effect of rTMS might be improved if patients could perform a cognitive task or therapy such as CCT during stimulation, resulting from the optimization of state-dependent effects. However, as this has not been studied before, a proof-of-principle study in healthy participants is needed to test this premise.

To further reinforce the state-dependent effects of rTMS, we aimed to precisely target a specific subregion of the left DLPFC. Recently, studies have investigated the use of resting-state connectivity-guided neuronavigation, which allows the identification of an individualized target within the DLPFC (Cole et al., 2018; Fitzgerald, 2021). In these approaches, a connectivity analysis is performed on individual resting-state fMRI data to identify the region within the DLPFC that is most strongly anti-correlated with the subgenual anterior cingulate cortex (sgACC) (Fox et al., 2012, 2013). Response to rTMS treatment can be predicted by distance to this optimal stimulation target, with patients receiving stimulation closer to this target showing better response (Cash et al., 2020).

In the current study, we aim to stimulate the DLPFC with rTMS while functionally engaging this area with CCT and to investigate whether this combination makes the DLPFC more receptive to the effects of stimulation with rTMS and could result in possible additive effects. We will use resting-state connectivity-guided neuronavigation to further strengthen the state dependency of rTMS as this allows for personalization of the DLPFC hub. We specifically want to assess the effect on mood and cognitive control, in a group of healthy participants. To measure the effect on mood, a negative mood is induced immediately after stimulation, as we know from our previous study that rTMS may affect the susceptibility to mood induction (Mobius et al., 2017). We hypothesize that the combination of active rTMS and CCT results in an increase in cognitive control and subsequently in a smaller decrease in mood after negative mood induction as compared to active rTMS combined with a control task. If the results are in line with this hypothesis, an additional comparison between active rTMS combined with CCT and sham rTMS combined with CCT will be performed.

## 2. Methods

### 2.1. Participants

Twenty healthy participants aged between 18 and 26 years were recruited. Participants were excluded if any of the following criteria applied: pregnancy; history of brain surgery; cardiac pacemaker or intracardiac lines; implanted neurostimulator; cochlear implants; a history of severe neurological problems such as epilepsy or severe head trauma; close relatives with epilepsy; a history of mood disorders; severe physical illness; metal in the cranium; or a score of ≥13 on the Beck Depression Inventory (BDI-II) (Beck et al., 1996).

### 2.2. Procedures

**Figure 1:**
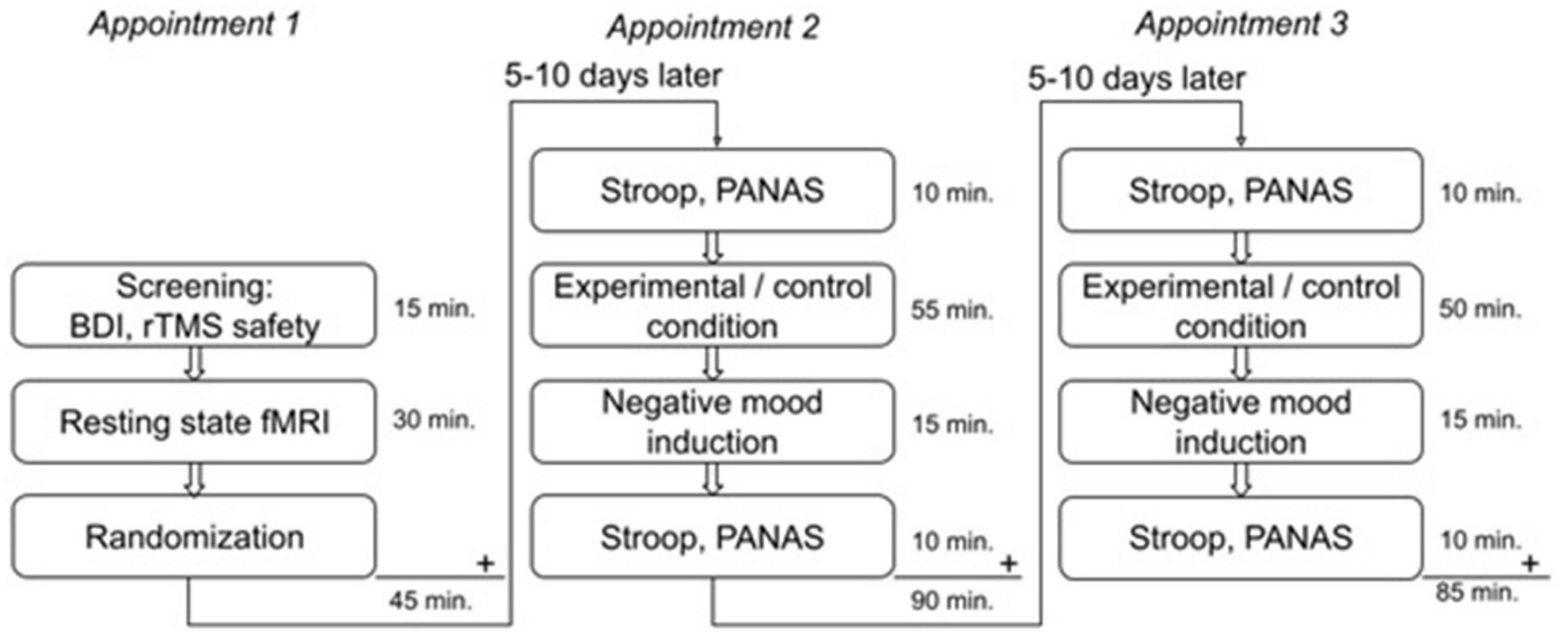
Overview of the study design including duration of each part of the experiment.

Participants were invited to the laboratory on three separate days, the appointments were scheduled 5–10 days apart. During the first appointment, participants were screened and MRI data were collected. Afterward, participants were randomized to determine the order of the conditions: experimental/control or control/experimental. Appointments 2 and 3 consisted of rTMS combined with either the experimental or control task, followed by negative mood induction. Participants also performed a Stroop task and filled in the Positive and Negative Affect Scale (PANAS). See Figure 1 for an overview of the study design. If after 20 participants a difference between conditions could be established, the pilot study would be extended. In this second part, active rTMS would be compared to sham rTMS, both in combination with the experimental task. See the Statistical Analysis section for a more detailed description.

### 2.3. Materials

Detailed information on all tasks can be found in the Supplementary material.

#### 2.3.1. Cognitive control training

In the experimental condition, participants performed a progressive dual n-back task (Jaeggi et al., 2007, 2008; Layden, 2018). Auditory and visual stimuli were presented sequentially. When a stimulus, either auditory or visual, was the same as n turns back (e.g., *n* = 2), participants needed to respond by pressing a key. The level of n was adapted based on their score. The control condition consisted of a single one-back task, where only visual stimuli were presented and n remained at one.

#### 2.3.2. Stroop task

This task was presented at the start of appointments 2 and 3 as well as after the negative mood induction, to assess cognitive control (Stroop, 1935; Vanderhasselt et al., 2009; Sivek, 2016). Words were presented in different colors, while the words themselves were the names of colors. Participants were asked to press a key corresponding to the color of the words as opposed to their names, as fast and accurately as possible. The task consists of congruent trials, where the colors and names match, and incongruent trials, where they do not match (e.g., “RED” written in blue). Based on the reaction time and number of errors, an interference score was calculated.

#### 2.3.3. Positive and negative affect scale

PANAS was used to assess mood at the start of appointments 2 and 3 and after the negative mood induction. It consists of 20 items, of which 10 assess positive affect and 10 assess negative affect (Watson et al., 1988).

#### 2.3.4. Negative mood induction

A 7-min clip from the movie “Sophie's Choice” was shown. This procedure was adapted from Fitzgerald et al. and has been used successfully in our laboratory (Fitzgerald et al., 2011; Mobius et al., 2017). Participants were instructed to empathize with the main protagonist. At the end of appointments 2 and 3, a 4-min clip from the movie “Jungle Book” was shown to negate the effects of mood induction before sending the participants home.

#### 2.3.5. Repetitive transcranial magnetic stimulation

For rTMS, a Magstim rapid2 with a double 70-mm air film coil was used. Motor threshold was determined as the minimal stimulation intensity at which a movement of the fingers or thumb was visually observed in ≥five of ten trials. Stimulation consisted of 2,400 pulses delivered at 10 Hz in 60 trains of 40 pulses each and an inter-train interval of 26 s. Neuronavigation was used to stimulate individualized targets within the DLPFC. This was performed using a Brainsight TMS neuronavigation system, v2.0 (Brainbox Ltd, Cardiff, UK).

#### 2.3.6. Functional magnetic resonance imaging

Scanning was performed using a 3T MAGNETOM Skyra MRI scanner (Siemens, AG, Healthcare Sector, Erlangen, Germany) and a 32-channel head coil. A T1-weighted MRI scan was acquired in the sagittal orientation for anatomical reference and analysis. This was carried out using an MPRAGE sequence with the following parameters: TR/TI = 2,300/1,100/3 ms, FA = 8°, FOV 256 × 256 × 192 mm, and a 1-mm isotropic resolution. Parallel imaging (iPAT = 2) was used to accelerate the acquisition. Participants were instructed to close their eyes. An 8-min resting-state fMRI scan was also acquired via single echo simultaneous multi-slice (MB) EPI with the following parameters: TR/TE = 1,000/35.2 ms, FA = 60°, FOV = 213 × 213 × 132 mm, slice number = 66, voxel size = 2.0 × 2.0 × 2.0 mm, and mb = 6. Participants were instructed to keep their eyes open and fixate on a white cross on a black screen while trying to relax.

The resting-state scan was analyzed by extracting the brain from the anatomical T1 images using the Brain Extraction Tool of FSL (FMRIB's Software Library, www.fmrib.ox.ac.uk/fsl). Distortions due to the phase encoding direction were corrected using a fieldmap image created by FSL's TOPUP tool based on 10 scans acquired using the inverse phase encoding direction. Data were then pre-processed with FEAT (fMRI Expert Analysis Tool). Registration to high-resolution and standard space images was carried out using FLIRT (Jenkinson and Smith, 2001; Jenkinson et al., 2002). Registration from high-resolution structural to standard space was refined using FNIRT non-linear registration (Andersson et al., 2007a,b). The functional scans were linearly registered to the T1 anatomical scan and non-linearly registered to the Montreal Neurological Institute (MNI) 152 T1 standard brain template. Using FEAT, the following pre-statistics processing was applied: motion correction using MCFLIRT (Jenkinson et al., 2002); brain extraction using BET (Smith, 2002); spatial smoothing using a full-width half-maximum (FWHM) Gaussian kernel of 3 mm; grand mean intensity normalization of the entire 4D dataset by a single multiplicative factor; and high-pass temporal filtering (Gaussian-weighted least-squares straight line fitting, with sigma = 50.0 s). Afterward, a bandpass filter (0.01–0.1 Hz) was applied to the data.

#### 2.3.7. Neuronavigation

A personalized left DLPFC target was determined for each participant according to the methods described previously (Beck et al., 1996; Mobius et al., 2017; Cash et al., 2020). The left DLPFC target was determined using the resting-state fMRI scan and calculating the region with the most anticorrelated functional connectivity with the right subgenual anterior cingulate cortex (sgACC).

**Figure 2:**
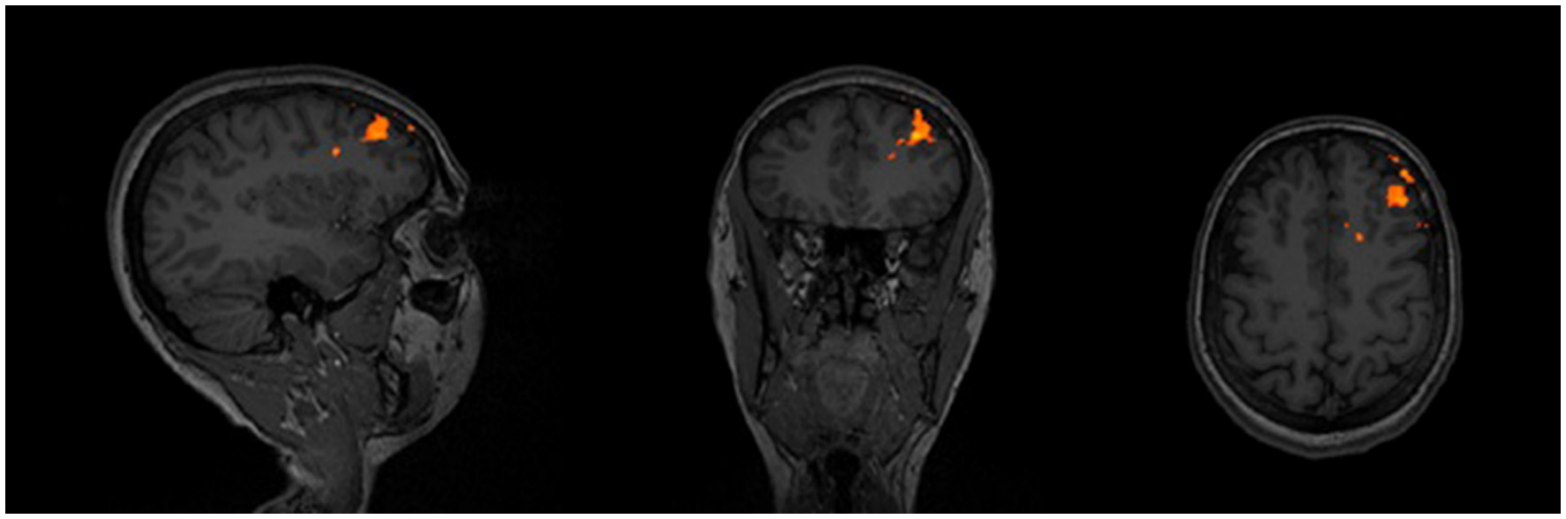
Example of the anticorrelation within the DLPFC, on which the neuronavigation target is based.

Region of interest (ROI) masks were created for two targets. The right sgACC mask (MNI 6,16,−10) was determined using a kernel sphere of 5 mm (Jing et al., 2002; Fox et al., 2013), whereas, for the left DLPFC (MNI −36,47,32), a kernel sphere of 25 mm was used (Tik et al., 2017). Both masks were transformed into subject space. An ROI analysis was performed on the preprocessed fMRI data using the right sgACC mask. Afterward, the anticorrelation was calculated between the extracted eigen variants of the sgACC ROI and the pre-processed whole-brain fMRI data of the subject. The resulting anticorrelation was mapped on the subjects' anatomical scan. Finally, the left DLPFC mask and a subject-specific threshold were applied resulting in the strongest anticorrelation within this region. This was used as the personalized DLPFC target during rTMS. See Figure 2 for an example.

### 2.4. Statistical analysis

A sample size calculation was performed using G^*^power (Faul et al., 2007), based on which the required sample size was estimated at *n* = 15 (see Supplementary material). To compensate for dropouts and potential overestimation of the effect, we aimed to include 20 participants.

After 20 participants, the results were analyzed. If the results of our primary outcome were in line with our hypothesis of a differential effect of CCT, defined as an effect size of *d* ≥ 0.2, then the pilot study would be extended by recruiting 20 more participants for the second part of the study. In this part, active rTMS would be compared to sham rTMS, both in combination with CCT.

All statistical analyses were performed using SPSS Statistics 22.0 (IBM Corp., Armonk, NY, USA), and procedures were two-tailed with significance set at an alpha-level of 0.05. Our primary outcome was the difference in PANAS scores from pre- to post-intervention between the experimental and control conditions. To assess this effect, a repeated-measures analysis of covariance (ANCOVA) was performed with time (pre-/post-intervention) and condition (experimental/control) as within factors and order of condition as a covariate. Our secondary outcome was the difference in the Stroop interference score from pre- to post-intervention, between the two conditions, which was assessed using repeated-measures ANCOVA with time (pre-/post-intervention) and condition (experimental/control) as within factors and order of condition as covariate. A paired *t*-test was performed on the pre- and post-measurements for each outcome measure, irrespective of condition or condition order, to check whether the negative mood induction was successful. Finally, a regression analysis was performed to assess the effect of change in the interference scores on change in the positive and negative PANAS scores.

## 3. Results

### 3.1. Participants

Twenty participants were included in this study. One participant was considered a screening failure (BDI-II score > 13), one was a no-show, and a third participant dropped out during the second appointment due to the rTMS being uncomfortable. Conform the sample size calculation which compensated for dropouts we did not replace subjects. The demographic characteristics of the 17 remaining participants are shown in Table 1. The Stroop scores of two participants had to be excluded because of invalidity, both from the control condition. An outlier on the positive PANAS scores was found in the experimental condition. Excluding this outlier normalized the distribution of the data.

**Table 1:** Demographic characteristics of participants.

	**Order 1 (*N* = 9)**	**Order 2 (*N* = 8)**	**Total (*N* = 17)**
Sex (m/f)	1/8	3/5	4/13
Age (years)	22.3 ± 2.6	22.1 ± 2.3	22.2 ± 2.4

Values represent N or mean ± SD. Order 1, experimental/control; Order 2, control/experimental.

### 3.2. Effect of negative mood induction

For an overview of the results of the paired *t*-test on pre- and post-measurements for each outcome measure independent of condition or condition order, see the Supplementary material.

The positive PANAS scores were higher before as opposed to after negative mood induction (M = 31.3, SD = 6.1; M = 25.4, SD = 7.3), which was significant [*t*(33) = 10.009, *p* < 0.001]. Negative PANAS scores also differed significantly between pre- and post-negative mood induction [*t*(33) = −4.610, *p* < 0.001], with lower scores before as opposed to after negative mood induction (M = 13.0, SD = 2.8; M = 17.0, SD = 5.1). Both results indicate that negative mood induction was successful.

The Stroop interference scores were higher before negative mood induction (M = 70.7, SD = 10.3) than after (M = 67.9, SD = 10.2). This difference was significant, *t*(31) = 2.259, *p* = 0.031, indicating that there was no deteriorating effect of negative mood induction after a single-session rTMS on interference, although we cannot rule out training effects on the Stroop task, which may mask this effect.

### 3.3. Effect on mood and cognitive control

The mean pre- and post-scores on the primary outcome measures are shown in Supplementary Table S2. There was no significant interaction between time and condition on the positive [*F*_(1, 15)_ = 0.019, *p* = 0.958] or the negative PANAS scores [*F*_(1, 15)_ = 0.626, *p* = 0.736]. Based on these results, the second part of the study was not performed. On the Stroop interference scores, no significant interaction between time and condition was found [*F*_(1, 13)_ = 15.419, *p* = 0.410].

**Figure 3:**
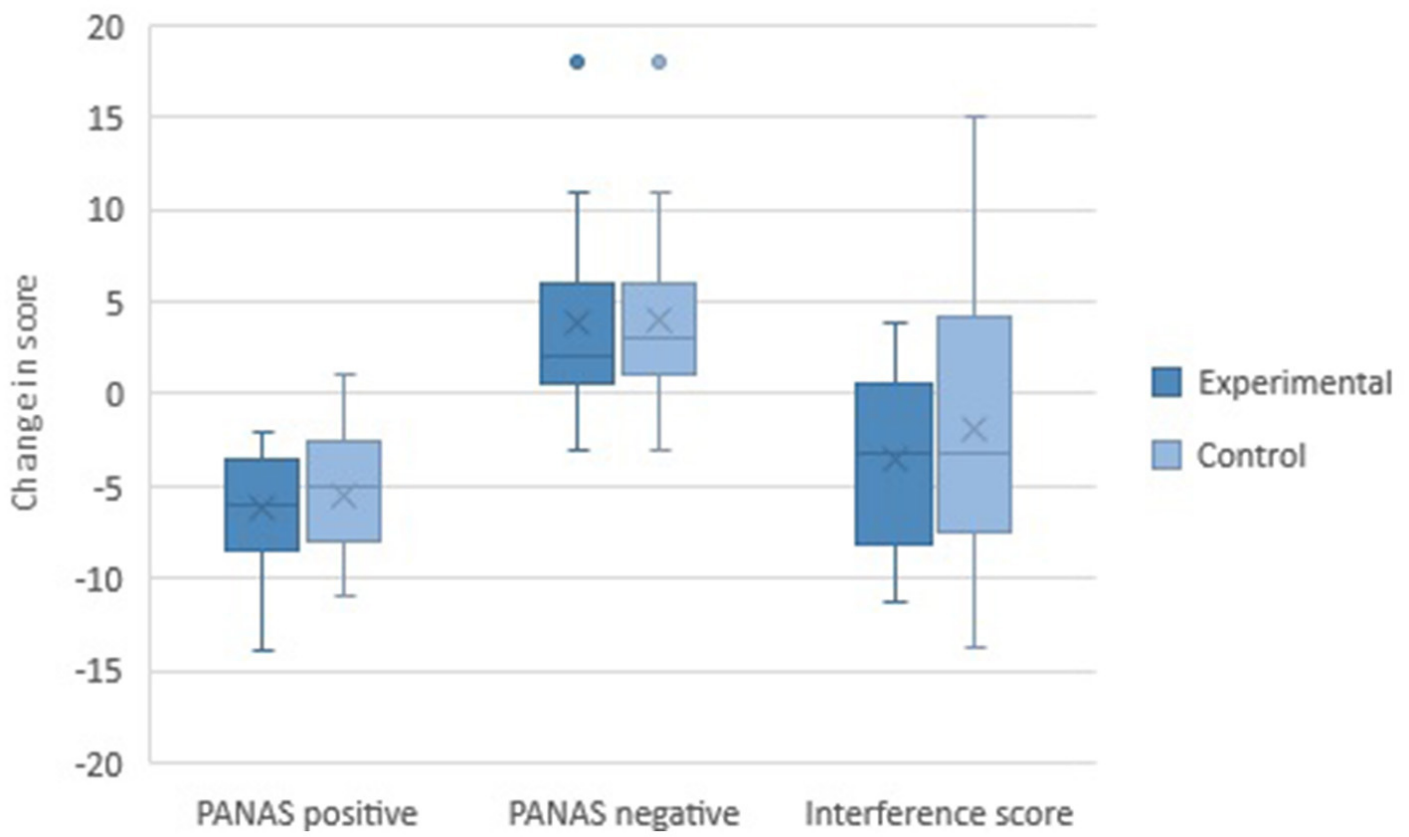
Mean change in outcome measures for each condition.

Figure 3 depicts the mean change in the interference score and the PANAS scores for each condition, indicating that the effects of negative mood induction were present in both conditions, with negative PANAS scores and interference scores increasing and positive PANAS scores decreasing. Overall, no difference in scores between the sessions with CCT and the sessions with the control task was observed. The change in the interference score and PANAS scores per condition order is shown in Supplementary Figure S3.

### 3.4. Relationship between the stroop interference score and PANAS scores

Regression analyses were performed on the effect of change in the Stroop interference score on the change in PANAS scores, correcting for baseline Stroop interference score. There was no significant relationship between the change in interference score and the change in positive PANAS score [*R*^2^ =.071, 0_(2, 29)_ = 1.110, *p* = 0.343]. Change in the Stroop interference score did not predict change in the positive PANAS score (β = −0.063, *p* = 0.744). No relationship was present for the effect of change in the interference score on the change in the negative PANAS score [*R*^2^ = 0.102, *F*_(2, 29)_ = 1.652, *p* = 0.209]. Change in the Stroop interference score did not predict change in the negative PANAS score (β = −0.320, *p* = 0.099).

### 3.5. Bayesian modeling

To check for the absence of an effect, a Bayesian approach was used (JASP, 2022). A Bayesian repeated-measures ANOVA for the positive PANAS scores revealed that the data are 6.384 times more likely under the null model than under the model including condition and condition order. For the negative PANAS scores, this value was 4.492. Finally, the Bayesian repeated-measures ANOVA for the Stroop interference scores indicated that the data are 6.323 times more likely under the null model as opposed to the model including condition and condition order. For the overview of all the results, see the Supplementary material.

## 4. Discussion

In this study, we investigated the potential additive effects of combining rTMS and CCT on mood and cognitive control in healthy participants by conducting a randomized within-subject pilot study incorporating individualized resting-state connectivity-guided neuronavigation. Our findings did not support our hypothesis as no significant effects were found on mood and cognitive control for the combination of rTMS and CCT as opposed to rTMS and a control task. Indeed, we found moderate evidence for the absence of an effect.

When comparing rTMS and CCT with rTMS and a control task, we did not find a differential effect on mood, irrespective of the order in which the conditions were presented. In both conditions, a decrease in positive affect and an increase in negative affect were observed as a function of the successful mood induction. These findings are in line with earlier studies from our laboratory, which showed that a single session of rTMS over the left DLPFC is not sufficient to change the negative mood in healthy individuals, although it can augment the effects of negative mood induction (Mobius et al., 2017; Bovy et al., 2019). Indeed, the ameliorating effects of rTMS on mood in depressed patients are seen after repeated sessions which might also be necessary to achieve effects in healthy participants.

Despite the negative mood induction, which we would expect to increase interference (Nixon et al., 2013), personalized neuronavigated rTMS combined with a task resulted in an improvement in interference, irrespective of whether this is a control task or a cognitively demanding task such as CCT. Whether rTMS is combined with a high- or low-load cognitive task is seemingly of no influence in healthy subjects. However, we also do not know whether rTMS on its own would have had the same effect or even the CCT or control task on its own. Nevertheless, we cannot rule out that the combination of rTMS with a cognitive task can be beneficial.

It is possible that the control task was efficient enough to overcome the interference, and there was no added value of the CCT. Another possible explanation could be the presence of a ceiling effect, as our sample consisted of young, healthy, and well-educated participants. As cognitive control declines with age, it may have been at too high a level in our young sample for the manipulation to have an effect (Stern, 2012; Lövdén et al., 2020). Stimulation of the DLPFC, and thus indirectly the networks involved in cognitive control, while functionally engaging these networks with CCT could have an additive effect that will only become apparent in individuals with impaired cognitive control.

Previous studies from our laboratory have also combined rTMS with negative mood induction or cognitive tasks (Mobius et al., 2017; Bovy et al., 2019). In the study of Möbius et al., it was investigated whether rTMS could protect against the effects of negative mood induction. Active rTMS induced a higher susceptibility to the mood induction procedure when this was carried out immediately following rTMS. We were unable to replicate this effect, although this could be the result of the differences in the mood induction procedure, as Möbius et al. used a short mood induction booster. The observed effects only became apparent after this booster. In contrast, in the study of Bovy et al. the order was reversed and rTMS was applied after negative mood induction. Furthermore, rTMS was combined with attentional bias modification (ABM), a cognitive task aimed at changing cognitive biases. As no effects on mood and attentional bias and control were observed, our findings are in line with these results. A single session of rTMS could be insufficient to induce differences in subtle cognitive processes such as attentional bias and mood. The current study further expanded on both studies using individual resting-state connectivity-guided neuronavigation, thereby improving on the previous studies by ensuring precise placement of the coil and accurate stimulation of personalized targets.

Transcranial direct current stimulation (tDCS) is a neuromodulation method that has been combined with CCT in depressed patients (Segrave et al., 2014). Patients were randomized into three groups (tDCS + CCT; sham tDCS + CCT, and tDCS + sham CCT) and received five sessions on consecutive working days. Although a decrease in depressive symptoms was seen in all three conditions, only the tDCS + CCT group showed sustained response at follow-up. As was the case in our study, no effect on cognitive control was observed. The results of this study emphasize that multiple sessions might be needed to find an effect, as well as multiple patient populations to prevent ceiling effects.

### 4.1. Strengths and limitations

The strength of the current study is the use of individual resting-state connectivity-guided neuronavigation, which allows for the integration of interindividual differences in functional connectivity, thereby personalizing the targeting of the DLPFC with rTMS and optimizing its effects (Fox et al., 2012; Fitzgerald, 2021). However, our study also has several limitations. First, our study consisted of young, healthy participants. A (sub-) clinical population might respond differently to our combined intervention, due to both the presence of depressive symptoms and potential differences in functional connectivity, as well as due to impairments in cognitive control that are often observed in depressed patients. A second limitation is that differences existed in the personalized neuronavigation targets. We observed that participants were better able to relax if the scan had more easily defined targets, whereas, in other participants, it was more difficult to properly determine the best target, which could have affected stimulation. Finally, we did not include a sham rTMS condition to separately assess the effects of CCT, although this was a conscious decision given that previous studies in our laboratory have examined the effects of active versus sham rTMS on mood and cognition (Mobius et al., 2017; Bovy et al., 2019).

### 4.2. Future directions

Future studies could improve on our design by including multiple rTMS sessions to assess whether the effects of rTMS that are seen in patients also translate to healthy individuals. Alternatively, our design could be conducted in a patient population, as rTMS and CCT are both safe and established treatments for depression. The addition of CCT to an rTMS session could be accomplished relatively easily, although the feasibility of performing this combination in a vulnerable population such as depressed patients needs to be considered. A clinical trial in which depressed patients are randomized to rTMS only or rTMS combined with CCT could establish whether the addition of CCT would add value to the existing rTMS protocol without risk to the patient.

## 5. Conclusion

Overall, no differential effect of a single session of rTMS combined with CCT was found on mood and cognitive control in healthy individuals, although a systemic effect was found of the combination of rTMS and a cognitive task on cognitive control. Furthermore, an effect of the negative mood induction was found, resulting in a decrease in positive affect and an increase in negative affect, which was irrespective of the condition. The use of resting-state connectivity-guided neuronavigation was a methodological improvement and could be valuable for clinical implementation.

## Data availability statement

The raw data supporting the conclusions of this article will be made available by the authors, without undue reservation.

## Ethics statement

The studies involving human participants were reviewed and approved by METC Oost-Nederland (NL77177.091.21). The patients/participants provided their written informed consent to participate in this study.

## Author contributions

ID: conceptualization, methodology, formal analysis, investigation, data curation, and writing—original draft. CS: software, formal analysis, investigation, data curation, and writing—original draft. BB: conceptualization, methodology, software, and writing—review and editing. KR, PE, and IT: conceptualization, methodology, and writing—review and editing. All authors contributed to the article and approved the submitted version.
