# Supplementary material for: Studying additive effects of combining rTMS with cognitive control training: a pilot investigation
Source: Front Hum Neurosci. 2023 Jul 31;17:1201344. doi: 10.3389/fnhum.2023.1201344 (PMC10423931; doi:10.3389/fnhum.2023.1201344)
Supplement: Supplementary file 1 [file Data_Sheet_1.PDF]

Supplementary Material

Studying additive effects of combining rTMS with cognitive control training, a pilot investigation

Iris Dalhuisen[1,2 *], Céline Schutte[1], Bob Bramson[2,3], Karin Roelofs[2,3], Philip van Eijndhoven[1,2], & Indira Tendolkar[1,2]

[1]Department of Psychiatry, Radboud University Medical Center, Nijmegen, The Netherlands

[2]Donders Institute for Brain Cognition and Behavior, Radboud University, Nijmegen, The Netherlands

[3]Behavioral Science Institute, Radboud University, Nijmegen, The Netherlands

*** Correspondence:**
Iris Dalhuisen
Iris.Dalhuisen@radboudumc.nl

1 Task details - CCT

For the experimental condition a progressive dual n-back task as described by Jaeggi et al. (1, 2) was used as cognitive control training. As is evident in supplementary figure S1,visuospatial and auditory stimuli were presented simultaneously to the participant. The participant had to remember the location of stimuli and auditory sounds and was supposed to respond when either the location or sound is the same as n-turns back, starting at n=2. If the participant scored ≥0.9 the level of the dual n-back was increased, to for instance n=3. If the score of the participant was ≤0.9 the n-back level was lowered. One block consisted of 20+n trials, each trial had a duration of 3000 ms. The visual spatial stimuli consisted of blue squares projected on a computer screen at 9 different location. The auditory stimuli consisted of eight consonants presented through a speaker. For experimental use in MATLAB the dual n-back task by Layden was used (3). See supplementary figure S1 for a visual representation of the task.

In the control condition a single n-back task was used. In this version the participants were only presented visual spatial stimuli and the n-back level was set at 1. There was no adjustment of the task based on performance.

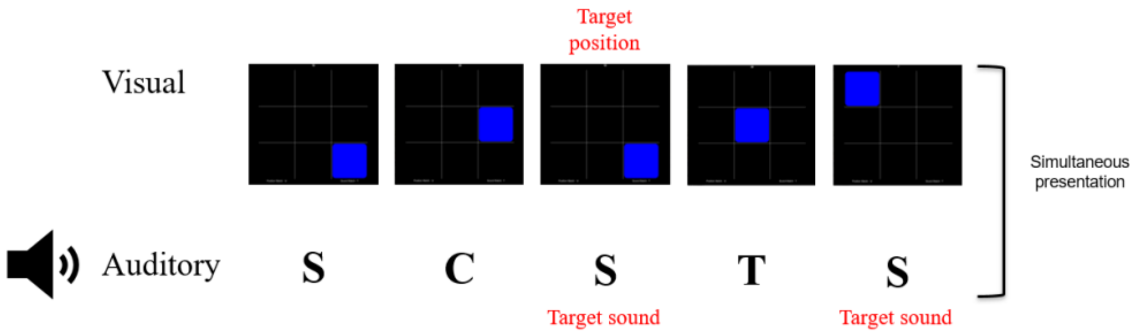

Figure S1. Visual representation of CCT using a progressive dual n-back task. In this example, n is set at 2.

2 Task details - Stroop

This interference score was calculated using the following formula;

Interference score = total score + ((2* mean reaction time per word) * number of uncorrected errors)

Total score = overall time for reading

Mean time per word = overall time for reading divided by the number of times

Number of uncorrected errors = the number of error not spontaneously corrected.

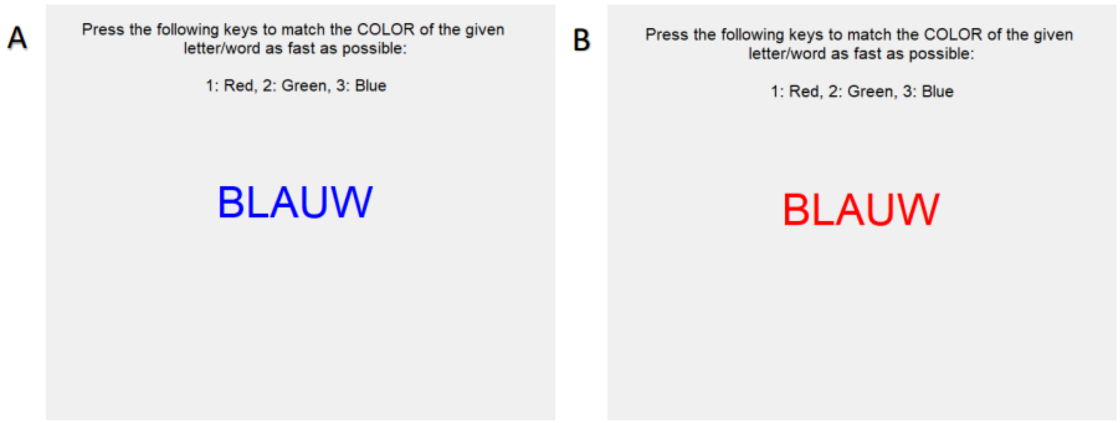

Figure S2. Experimental presentation of the adapted Stroop task in Matlab. A. Congruent condition. B. Incongruent condition.

3 Sample size calculation

The sample size calculation is based on previous studies on similar subjects, as this exact paradigm has not been tested before. A study performed by our group investigated the effect of rTMS on negative mood induction and found an effect size of d = 0.81 (4). When doing a sample size calculation using G*power (5), this resulted in a sample size estimation of N = 15. See table 1 for the input and output parameters. Another study assessed the effect of CCT on NMI, and reported an effect size of d = 0.89 (6). Using this effect size, the sample size calculation resulted in N = 12 (input and output parameters can be found in table 1).

Based on this, we attempted to include at least 15 participants. To compensate for drop-outs and for a potential

l overestimation of the effect, we included a total of 20 participants.

Table S1. Input and output parameters of sample size calculation using G*power.

		**Effect size *d* = 0.81**	**Effect size *d* = 0.89**
**Input parameters**	Test	ANOVA, repeated measures, within factors	ANOVA, repeated measures, within factors
	Effect size *f*	0.405 (Cohen's *d* = 0.81, converted to *f* = 0.405)	0.445 (Cohen's *d* = 0.89, converted to *f* = 0.445)
	Alpha error probability	0.05	0.05
	Power	0.80	0.80
	Number of groups	1	1
	Number of measurements	2	2
	Correlation among repeated measures	0.5	0.5

	Nonspheriticy correction	1	1
**Output parameters**	Noncentrality parameter	9.84	9.51
	Critical F	4.60	4.84
	Numerator df	1	1
	Denominator df	14	11
	Total sample size	15	12
	Actual power	0.83	0.80

4 Analyses

Table S2. Mean pre- and post-scores on Stroop and PANAS, per condition and per order.

		**Order 1 (N = 9)**		**Order 2 (N = 8)**		**Total (N = 17)**	
		pre	post	pre	post	pre	Post
**Exp.**	PANAS positive	31.8±6.5	25.4±6.2	30.6±7.6	24.6±8.4	31.2±6.9	25.1±7.1
	PANAS negative	13.6±2.7	18.7±6.8	12.6±2.8	15.0±2.8	13.1±2.7	16.9±5.5
	Interference	70.9±8.3	66.9±8.0	68.4±16.4	65.3±12.7	69.7±12.4	66.2±10.1
**Cont.**	PANAS positive	30.4±5.5	24.4±7.6	32.4±5.4	27.3±8.0	31.4±5.4	25.8±7.7
	PANAS negative	13.2±3.8	18.1±6.1	12.6±1.8	15.8±3.0	12.9±2.9	17.0±4.9
	Interference	73.3±9.9	72.4±12.8	69.9±4.1	67.0±6.4	71.7±7.7	69.9±10.3

Values represent mean ± SD. Order 1 = experimental - control. Order 2 = control – experimental.

Results of the mixed ANOVA on positive PANAS score, negative PANAS score, and interference score, with the outliers excluded. For each analysis, order was the between factor and condition (experimental / control) the within factor.

Results of the paired t-tests on the effect of negative mood induction on positive PANAS score, negative PANAS score, and interference score.

Table S3. Paired t-test results.

	**Pre-NMI scores**	**Post-NMI scores**	**p**
**PANAS positive**	31.3 ± 6.1	25.4 ± 7.3	<.001*
**PANAS negative**	13.0 ± 2.8	17.0 ± 5.1	<.001*
**Interference**	70.1 ± 10.3	67.9 ± 10.2	.031*

NMI = negative mood induction. Values represent mean ± SD. * indicates a statistically significant result.

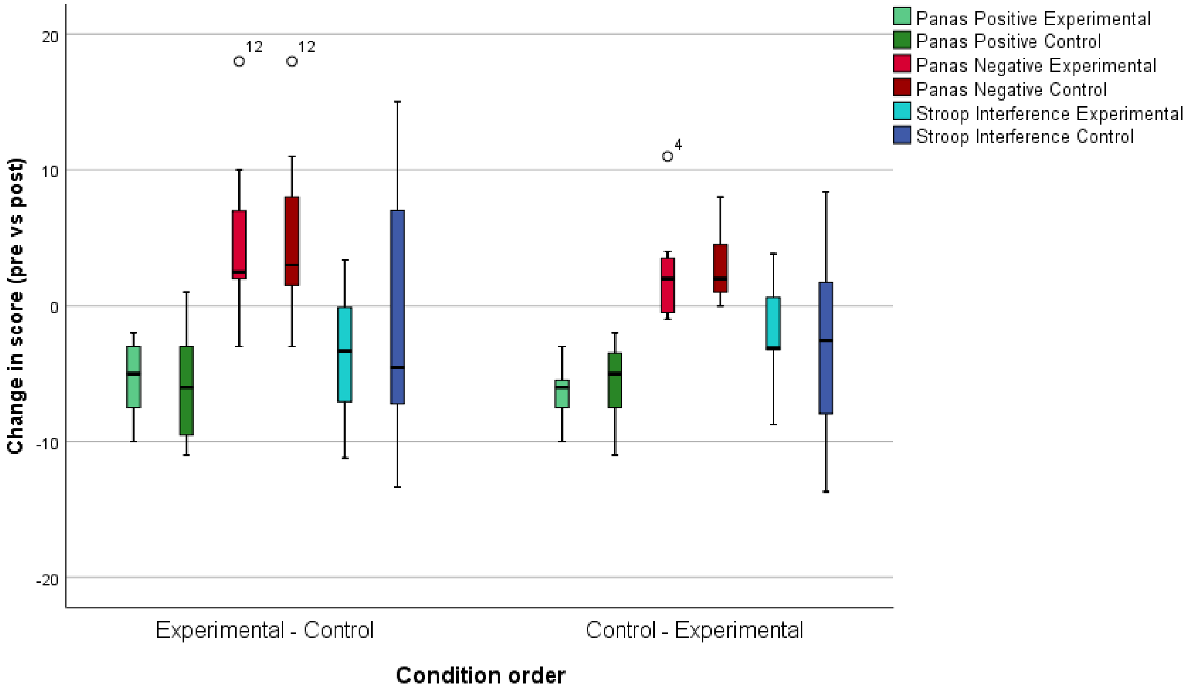

Figure S3. Mean change in outcome measures per condition order. Order 1 = experimental - control , order 2 = control – experimental.

5 Bayesian analyses

Results of the Bayesian repeated measures ANOVA, performed with JASP (7).

5.1 Bayesian repeated measures ANOVA – positive PANAS scores

Table S5. Bayesian repeated measures ANOVA model comparison for positive PANAS scores.

Models	P(M)	P(M|data)	BFM	BF01	error %
Null model (incl. subject and random slopes)	0.200	0.494	3.910	1.000	
Order	0.200	0.215	1.095	2.300	1.584
PANAS positive	0.200	0.181	0.884	2.732	1.384
PANAS positive + Order	0.200	0.077	0.336	6.384	1.911
PANAS positive + Order + PANAS positive ✻ Order	0.200	0.032	0.134	15.260	2.484

Note. All models include subject, and random slopes for all repeated measures factors.

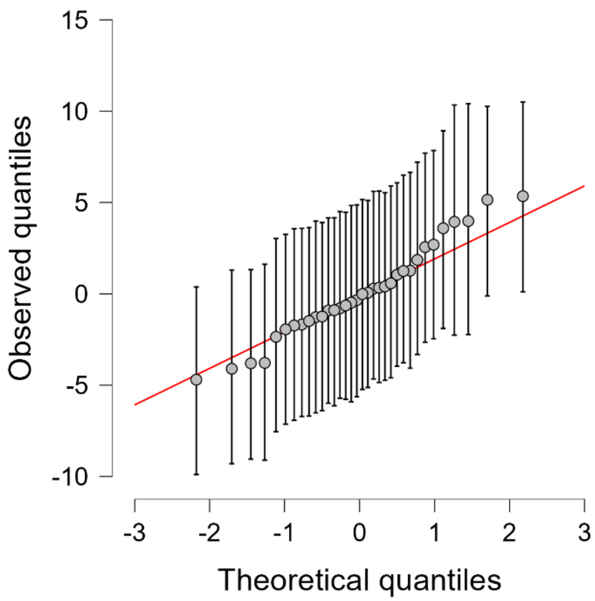

Figure S4. Q-Q plot for positive PANAS scores.

Table S6. Descriptives of positive PANAS scores for experimental and control condition and condition order separately.

							95% Credible Interval	
PANAS positive	Order	N	Mean	SD	SE	Coefficient of Variation	Lower	Upper
Experimental	1	9	-6.333	3.937	1.312	-0.622	-9.360	-3.307
	2	8	-6.000	2.507	0.886	-0.418	-8.096	-3.904
Control	1	9	-6.000	4.031	1.344	-0.672	-9.099	-2.901
	2	8	-5.125	3.441	1.217	-0.671	-8.002	-2.248

5.2 ayesian repeated measures ANOVA – negative PANAS scores

Table S7. Bayesian repeated measures ANOVA model comparison for negative PANAS scores.

Models	P(M)	P(M|data)	BFM	BF01	error %
Null model (incl. subject and random slopes)	0.200	0.427	2.978	1.000	
Order	0.200	0.292	1.648	1.463	1.371
PANAS negative	0.200	0.143	0.668	2.982	2.132
PANAS negative + Order	0.200	0.095	0.420	4.492	1.697
PANAS negative + Order + PANAS negative ✻ Order	0.200	0.043	0.181	9.839	2.007

Note. All models include subject, and random slopes for all repeated measures factors.

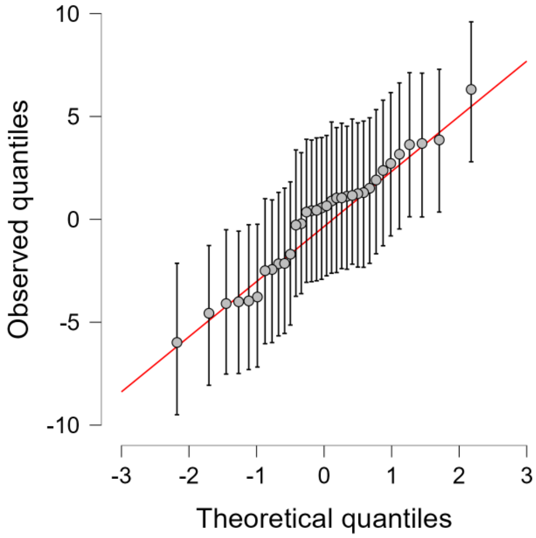

Figure S5. Q-Q plot for negative PANAS scores.

Table S8. Descriptives of negative PANAS scores for experimental and control condition and condition order separately.

							95% Credible Interval	
PANAS negative	Order	N	Mean	SD	SE	Coefficient of Variation	Lower	Upper
Experimental	1	9	5.111	6.112	2.037	1.196	0.413	9.809
	2	8	2.375	33.926	1.388	1.653	-0.907	5.657
Control	1	9	4.889	6.194	2.065	1.267	0.128	9.650
	2	8	3.125	2.949	1.043	0.944	0.660	2.290

5.3 Bayesian repeated measures ANOVA – Stroop interference scores

Table S9. Bayesian repeated measures ANOVA model comparison for interference scores

Models	P(M)	P(M|data)	BFM	BF01	error %
Null model (incl. subject and random slopes)	0.200	0.486	3.777	1.000	
Order	0.200	0.221	1.136	2.196	2.022
Interference score	0.200	0.173	0.838	2.805	0.764
Interference score + Order	0.200	0.077	0.333	6.323	1.068
Interference score + Order + Interference score ✻ Order	0.200	0.043	0.181	11.247	1.697

Note. All models include subject, and random slopes for all repeated measures factors.

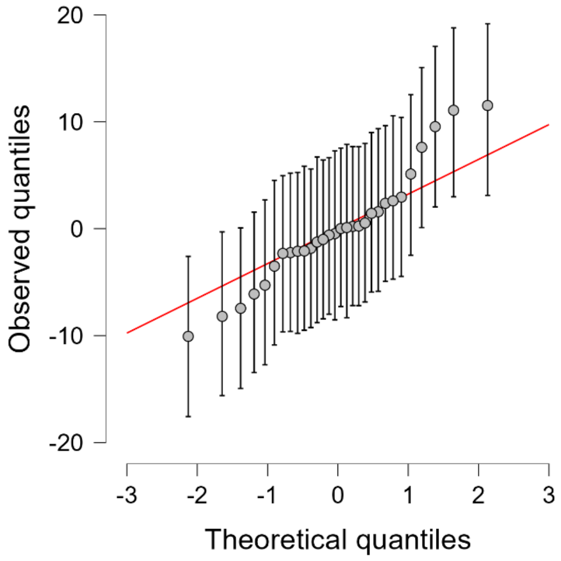

Figure S6. Q-Q plot for Stroop interference scores.

Table S10. Descriptives of interference scores for experimental and control condition and condition order separately.

Interference score	Order	N	Mean	SD	SE	Coefficient of Variation	95% Credible Interval Lower	95% Credible Interval Upper
Experimental	1	8	-3.607	5.015	1.773	-1.390	-7.799	0.586

							95% Credible Interval	
Interference score	Order	N	Mean	SD	SE	Coefficient of Variation	Lower	Upper
	2	7	-1.899	4.051	1.531	-2.1333	-5.645	1.847
Control	1	8	-0.964	10.307	3.644	-10.687	-9.582	7.6553
	2	7	-2.912	7.832	2.960	-2.690	-10.156	4.332

6 References

1. Jaeggi SM, Buschkuehl M, Etienne A, Ozdoba C, Perrig W, Nirkko A. On how high performers keep cool brains in situations of cognitive overload. Cognitive, affective & behavioral neuroscience. 2007;7:75-89.

2. Jaeggi SM, Buschkuehl M, Jonides J, Perrig WJ. Improving fluid intelligence with training on working memory. Proceedings of the National Academy of Sciences. 2008;105(19):6829-33.

3. Layden E. N-Back for Matlab. G-Node; 2018.

4. Mobius M, Lacomble L, Meyer T, Schutter D, Gielkens T, Becker ES, et al. Repetitive transcranial magnetic stimulation modulates the impact of a negative mood induction. Social cognitive and affective neuroscience. 2017;12(4):526-33.

5. Faul F, Erdfelder E, Lang A-G, Buchner A. G*power 3: A flexible statistical power analysis program for the social, behavioral, and biomedical sciences. Behavior Research Methods. 2007;39:175-91.

6. Calkins AW, Deveney CM, Weitzman ML, Hearon BA, Siegle GJ, Otto MW. The effects of prior cognitive control task exposure on responses to emotional tasks in healthy participants. Behav Cogn Psychother. 2011;39(2):205-20.

7. JASP (Version 0.16.4) [Computer software]. JASP team; 2022.

For more information on Supplementary Material and for details on the different file types accepted, please see here.

Supplementary Figures

Supplementary Figure 1. The figure legends are required to have the same font as the main text, 12 point normal Times New Roman, single spaced. Please use a single paragraph for each legend and prepare the figures keeping in mind the PDF layout.
